# Non-mono-exponential diffusion models for assessing early response of liver metastases to chemotherapy in colorectal Cancer

**DOI:** 10.1186/s40644-019-0228-2

**Published:** 2019-06-19

**Authors:** Yang Zhou, Hong-Xia Zhang, Xiu-Shi Zhang, Yun-Feng Sun, Kuang-Bang He, Xi-Qiao Sang, Yue-Min Zhu, Zi-Xiang Kuai

**Affiliations:** 1 Imaging Center, Harbin Medical University Cancer Hospital, Haping Road No.150, Nangang District, Harbin, 150081 China; 2 Division of Respiratory Disease, The Fourth Hospital of Harbin Medical University, Harbin, 150001 China; 3 CREATIS, CNRS UMR 5220-INSERM U1206, University Lyon 1-INSA Lyon-University Jean Monnet Saint-Etienne, 69621 Lyon, France

**Keywords:** Non-mono-exponential, Diffusion-weighted imaging, Chemotherapy, Liver metastasis, Colorectal cancer

## Abstract

**Background:**

Preoperative chemotherapy is becoming standard therapy for liver metastasis from colorectal cancer, so early assessment of treatment response is crucial to make a reasonable therapeutic regimen and avoid overtreatment, especially for patients with severe side effects. The role of three non-mono-exponential diffusion models, such as the kurtosis model, the stretched exponential model and the statistical model, were explored in this study to early assess the response to chemotherapy in patients with liver metastasis from colorectal cancer.

**Methods:**

Thirty-three patients diagnosed as colorectal liver metastasis were evaluated in this study. Diffusion-weighted images with b values (0, 200, 500, 1000, 1500, 2000 s/mm^2^) were acquired at 3.0 T. The parameters (*ADC*_*k*_*, K, DDC,α, D*_*s*_ *and σ*) were derived from three non-mono-exponential models (the kurtosis, stretched exponential and statistical models) as well as their corresponding percentage changes before and after chemotherapy. The difference in above parameters between the response and non-response groups were analyzed with independent-samples T-test (normality) and Mann–Whitney U-test (non-normality). Meanwhile, receiver operating characteristic curve (ROC) analyses were performed to assess the response to chemotherapy.

**Results:**

Significantly lower values of *K* (the kurtosis coefficient derived from the kurtosis model) and *σ* (the width of diffusion coefficient distribution in the statistical model) (*P* < 0.05) were observed in the respond group before treatment, as well as higher Δ*K* and Δ*σ* values (*P* < 0.05) after the first cycle of chemotherapy were also found compared with the non-respond group. ROC analyses showed the *K* value acquired before treatment had the highest diagnostic performance (0.746) in distinguishing responders from non-responders. Furthermore, the high sensitivity (100%) and accuracy (76.3%) from the *K* value before treatment was found in assessing the response of colorectal liver metastasis to chemotherapy.

**Conclusions:**

The non-mono-exponential diffusion models may be able to predict early response to chemotherapy in patients with colorectal liver metastasis.

## Background

Colorectal cancer (CRC) is the second most common cause of cancer-related mortality, and liver metastases are present in approximately 20% of the CRC patients [1]. Neoadjuvant chemotherapy followed by residual tumor excision is becoming the standard of care for liver metastases [2]. For unresectable liver metastasis, neoadjuvant chemotherapy can also decrease tumor loading and improve survival quality [3]. However, not all patients would respond to chemotherapy. The non-responders not only do not benefit from chemotherapy, but also might suffer side effects caused by chemotherapy [4]. Therefore, early evaluation of chemotherapy response is crucial for therapeutic strategy adjustment and preventing unnecessary medical interventions for the non-responders.

Traditional criteria for evaluating the chemotherapy response of liver metastasis are based on tumor size [5, 6]. But there is a growing consensus that the morphology evaluation exists limitation for relatively late changes in tumor size induced by chemotherapy [7]. Furthermore, the structural and functional changes in tumor microenvironment usually precede macroscopic morphological changes for chemotherapy. So traditional criteria have inherent weaknesses in early assessment of treatment response.

Diffusion-weighted (DW) imaging (DWI), combined with the apparent diffusion coefficient (*ADC*), can be used to reflect changes in tumor microenvironment during treatment in some extent [8–10]. Some studies have reported that *ADC* can predict early tumor response to chemotherapy [11–13]. However, *ADC* value was calculated by a mono-exponential relationship between the DW signal and *b* value based on tumor homogeneity and Gaussian movement of water molecules [14]. However, the diffusion behavior of water molecules in tumors is rather complicated or non-Gaussian, so recent studies suggests that using mono-exponential model to describe DW signal decay might be inappropriate [15–17]. Especially, when *b*-value exceeds a certain threshold (e.g. *b* = 1000 s/mm^2^), the DW signal deviates gradually from the mono-exponential decay for the tumor heterogeneity [18, 19]. Thus, some new non-mono-exponential diffusion models, such as the kurtosis [20], stretched exponential [21] and statistical models [22], were proposed to characterize the tumor heterogeneity. In contrast to the mono-exponential model, these non-mono-exponential models can provide more diffusion-related parameters and give additional information on the properties of tumor [15]. Therefore, non-mono-exponential models were expected as a new method to assess treatment response of tumors in early phase with good performance.

Although previous studies have attempted to use the non-mono-exponential models to evaluate the efficacy of chemotherapy [19, 23, 24], assessment of early chemotherapy response of colorectal liver metastases using these models has not yet been reported. Therefore, the aim of this study was to investigate the roles of the non-mono-exponential models in early assessment of the chemotherapy response of colorectal liver metastasis.

## Methods

### Patients

This prospective study was conducted under the approval of the local institutional research ethics committee and informed consent was obtained from each patient. From February 2018 to August 2018, 41 patients who satisfied the inclusion criteria underwent customized MRI examination for this study. Inclusion criteria were that the patient must be diagnosed with colorectal liver metastasis and that the patient did not previously receive any local or systemic chemotherapy or radiotherapy. Eight patients were excluded for the following reasons: i) unsatisfactory tumor position, for example, the top-left liver lobe where the DW images usually present artifacts due to cardiac motion, (*n* = 1), ii) poor image quality that interfered with the delineation of tumors (*n* = 3), or iii) loss of follow-up (*n* = 4). Thus, 33 patients, including 19 males (a mean age of 56.7 years; range from 37 to 77 years) and 14 females (a mean age of 54.6 years; range from 39 to 67 years) were finally enrolled in this study.

The diagnosis of 20 patients was proved by biopsy or surgical resection. For the rest of patients (*n* = 13), the diagnosis was were established by typical MRI features of liver metastasis: 1) irregular or ill-defined borders with low T1 signal intensity (SI) and variable high T2 SI; 2) peripheral rim enhancement; 3) relatively hypo-enhancement on portal or delayed phase in comparison with liver parenchyma; 4) interval growth of at least 20% in the longest axial diameter on serial cross-sectional imaging.

All enrolled patients received a 2-week cycle of administration of CAPEOX and were treated with four to six cycles according to their treatment response and physical situation. CAPEOX was consisted of a 2-h intravenous infusion of oxaliplatin 130 mg/m^2^ on day 1 plus oral administration of capecitabine 1000 mg/m^2^ twice daily for 14 days. Baseline of MRI was performed on 1 day before start of the first cycle of chemotherapy. Posttreatment MRI was performed 4~6 days after completion of the first cycle.

### MRI examination

All MRI examinations were conducted on a 3.0 T clinical whole-body MR imaging system (Ingenia, Philips Medical Systems, Eindhoven, The Netherlands) with a 32-channel phased-array coil. Patients were asked to fast for more than 5 h before scanning.

The scan protocol was consisted of the routine MRI sequences and a DWI sequence.

The routine MRI sequences included (a) an axial fat-suppressed T2-weighted turbo spin echo (TSE) sequence (TR/ TE 535/75 ms, slice thickness 7 mm, slice gap 1 mm, matrix size 232 × 199, FOV 350 mm × 392 mm), (b) coronal T2-weighted TSE sequence (TR/TE 1100/80 ms, slice thickness 6 mm, slice gap 1 mm, matrix size 292 × 253, FOV 350 mm × 346 mm), and (c) axial breath-hold dual-echo T1-weighted fast field-echo sequence (TR/TE1/TE2 106/1.15/2.3 ms, slice thickness 7 mm, slice gap 1 mm, matrix size 244 × 181, FOV 400 mm × 322 mm).

The DWI images were acquired using a spin-echo EPI sequence with gated-navigator respiratory motion compensation technique (the navigator gating window was set as 5 mm). Six *b* values (0, 200, 500, 1000, 1500, 2000 s/mm^2^ with 1, 1, 2, 2, 2, 4 signal averages, respectively) were applied in three orthogonal diffusion encoding directions [25]. The imaging parameters were: TR/TE 2000 /70 ms, number of slices 24, slice thickness 7 mm, slice gap 0 mm, matrix size 148 × 148, FOV 400 mm × 400 mm, NSA 2. The total scan time of the routine MRI sequences and DWI sequence was 24~27 min for different patients. Additionally, the patients who did not have pathologically confirmed hepatic metastases underwent a dynamic enhanced MR examination before the DWI scan for diagnosis.

The scan protocol was consisted of above routine MR sequences and a dynamic 3D breath-hold T1-weighted sequence with fat suppression. The dynamic enhanced MR imaging parameters were as follows: TR/TE1/TE2 3.6/1.32/2.3 ms, slice thickness 5 mm, slice gap 2.5 mm, matrix size 200 × 250, FOV 320 mm × 427 mm. The arterial, portal venous equilibrium and delayed phase were set as 20, 55, 90, and 180 s after contrast media injection, respectively.

### Image analysis

All DW images obtained at pre-treatment and post-treatment (i.e. 4~6 days after completion of the first chemotherapy cycle) were independently reviewed by two radiologists with 15 and 7 years’ experience in reading MR images, respectively. They were all blinded to the final therapeutic response. For the pretreatment images, the largest tumor area slice of each patient was selected. On the slice, one or more regions of interest (ROIs) (if existing metastases) were created manually by each observer to cover as much of the solid part of tumors as possible and to avoid surrounding necrosis, hemorrhagic areas, vessels or bile ducts. As for the images after treatment, the slices with the same position of those before treatment were drawn in same way.

The ROIs were initially delineated on b0 image, then copied and pasted on corresponding DW images. The DW images were normalized by dividing them by the b0 images, and geometric mean of these images over different diffusion encoding directions was taken [25]. For each patient, the voxel-level DW data on ROIs were fitted by the non-mono-exponential diffusion models and the median values of calculated parameters over all ROIs were recorded as the measurements of the patient.

Above measurements were executed twice by observer I and were executed once by observer II regardless of pretreatment or posttreatment. The two executions for observer I was to evaluate intra-observer reproducibility. The assessment of inter-observer reproducibility was performed between the first measurement of observer I and the measurement of observer II. For each patient, the average of first measurements record by observer I and measurements recorded by observer II was calculated and taken as the final measurements of the patient.

The non-mono-exponential diffusion models are as follows:

1. Kurtosis model [20]

1 $$ \frac{S(b)}{S_0}={e}^{\left(-{bADC}_k+\frac{1}{6}{b}^2{ADC}_k^2K\right)} $$

2. Stretched exponential model [21]

2 $$ \frac{S(b)}{S_0}={e}^{-{(bDDC)}^{\alpha }} $$

3. Statistical model [22]

3 $$ \frac{S(b)}{S_0}=\left(\frac{1+\phi \left(\frac{D_s}{\sigma \sqrt{2}}+\frac{b\sigma}{\sqrt{2}}\right)}{1+\phi \left(\frac{D_s}{\sigma \sqrt{2}}\right)}\right){e}^{\left(-{b D}_s+\frac{1}{2}{b}^2{\sigma}^2\right)} $$

where *S*(*b*) is the signal intensity for a givenvalue, *S*_0_ is *S* (*b* = 0 s/mm^2^), *ADC*_*k*_ and *K* stands for respectively the apparent diffusion coefficient and the kurtosis coefficient of the kurtosis model, *DDC* and *α* is the distributed diffusion coefficient and the anomalous exponent term of the stretched exponential model, *D*_*s*_ and*σ* represents respectively the peak position and the width of diffusion coefficient distribution in the statistical model. The nonlinear regression is implemented using the Levenberg–Marquardt algorithm in Matlab (version R2014a, Mathworks, Natick, MA).

Percentage changes in the diffusion-related parameters (i.e. *ADC*_*k*_, *K*, *DDC*, *α*, *D*_*s*_ and *σ*) were calculated according to, where Pre-*para* and Post-*para* refer to the values of parameters before and after treatment, respectively.

### Response evaluation

The response evaluation was performed on computed tomography (CT) between baseline and after 3 months from the start of the first cycle of chemotherapy. The overall response was determined according to Response Evaluation Criteria in Solid Tumors (RECIST) version 1.1 [26]. For patients with multiple metastases, the diameters of a maximum of two metastases in the liver were measured and summed to compare the total of those lesions’ diameters at baseline and after 3 months from the start of the first cycle of chemotherapy. Complete response (CR): no residual tumor was observed; partial response (PR): at least a 30% decrease was observed in the sum of the longest lesion diameters in comparison with the primary tumor size; progressive disease (PD): 20% or more increase in the sum of the longest diameters; and stable disease (SD): neither sufficient shrinkage to qualify for PR nor sufficient increase to qualify for PD. Patients who presented CR or PR were classified as responders, and patients who presented SD or PD were classified as non-responders.

### Statistical analysis

All parameter values were expressed as means ± standard deviations. The difference of measured parameter values between intra- or inter-observers was assessed using the Mann-Whitney U test and the intraclass correlation coefficient (ICC) was calculated to evaluate intra- and inter-observer reproducibility. The repeatability of parameters was defined as excellent when ICC values were between 0.75~1, good when ICC values were between 0.60~0.74, fair when ICC values were between 0.40~0.59, and poor when ICC values were smaller than 0.4 [27]. The differences above diffusion-related parameters and their percentage changes before and after chemotherapy between response and non-response groups were assessed using independent-samples T-test (normality) and Mann–Whitney U-test (nonnormality). The diagnostic accuracy of indicators (Pre-*K*, Δ*K*, Post-*σ*, and Δ*σ*) was evaluated in terms of sensitivity, specificity, positive predictive value (PPV), negative predictive value (NPV), and area under the receiver operating characteristic (ROC) curve (AUC). Their cutoff values were determined using the maximum Youden’s Method. All statistical analyses were conducted using commercial software (SPSS, v25.0, Chicago, USA; Medcalc, v11.4, Mariakierke, Belgium). Statistical significance was considered for *P* < 0.05.

## Results

### Patient characteristics

**Fig. 1:**
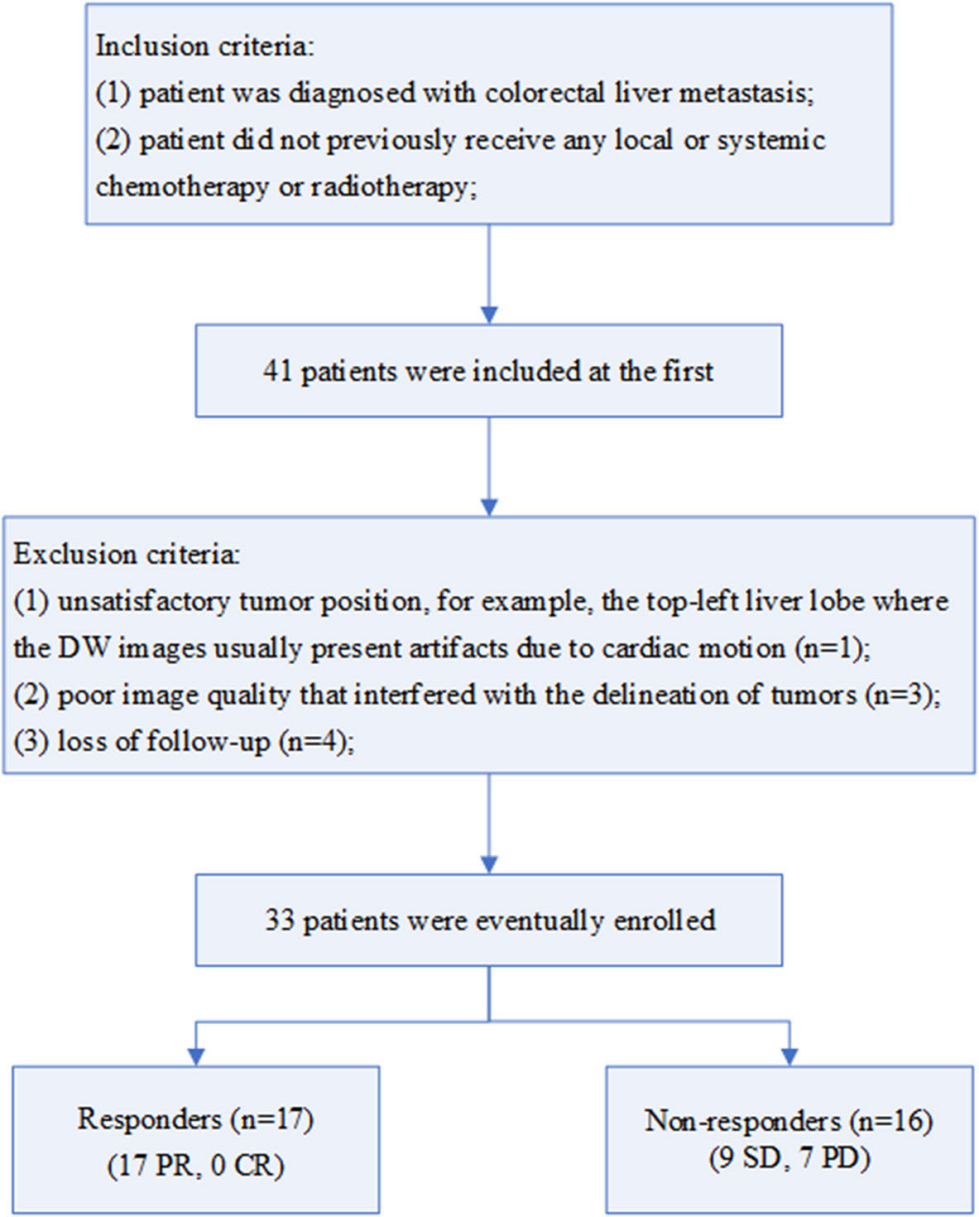
Flow chart of the study population as well as the responders and non-responders

We successfully calculated the parameter values from the three non-mono-exponential models for the 33 enrolled patients in both the pretreatment and early treatment periods. The response group was composed of 17 patients with PR (no one with CR). The non-response group was composed of remaining 16 patients (9 SD and 7 PD). The flow chart of the study population as well as the responders and non-responders, was shown as Fig. 1.

### Intra- and interobserver repeatability

No significant differences between intra- and inter-observer were observed in the diffusion-related parameters derived from the non-mono-exponential models (Table 1). All parameters showed excellent or good reproducibility.

**Table 1:** Intra- and Inter-observer agreement (measured as the ICC) for the diffusion-related parameters measured before and after chemotherapy

	Parameters	Intra-observer ICC(95%CI)	*P*-value	Inter-observer ICC(95%CI)	*P*-value
Pre-treatment	*ADC*_*k*_	0.914 (0.834~0.9456)	0.783	0.804 (0.639~0.898)	0.914
	*K*	0.929 (0.861~0.964)	0.893	0.717 (0.500~0.850)	0.453
	*DDC*	0.948 (0.898~0.974)	0.913	0.706 (0.485~0.842)	0.207
	*α*	0.918 (0.841~0.959)	0.788	0.877 (0.767~0.937)	0.453
	*D*_*s*_	0.937 (0.877~0.968)	0.964	0.887 (0.784~0.942)	0.903
	*σ*	0.924 (0.852~0.961)	0.863	0.816 (0.659~0.904)	0.631
Post-treatment	*ADC*_*k*_	0.955 (0.910~0.977)	0.842	0.811 (0.650~0.902)	0.715
	*K*	0.932 (0.867~0.965)	0.734	0.874 (0.762~0.936)	0.724
	*DDC*	0.937 (0.877~0.968)	0.744	0.798 (0.630~0.895)	0.256
	*α*	0.853 (0.724~0.924)	0.547	0.824 (0.637~0.914)	0.314
	*D*_*s*_	0.914 (0.834~0.957)	0.658	0.804 (0.639~0.898)	0.893
	*σ*	0.948 (0.899~0.974)	0.852	0.708 (0.486~0.844)	0.842

*ICC* inter-class correlation coefficient, *ADC*_*k*_ apparent diffusion coefficient from the kurtosis model, *DDC* distributed diffusion coefficients

### Parameters before and after chemotherapy

**Fig. 2:**
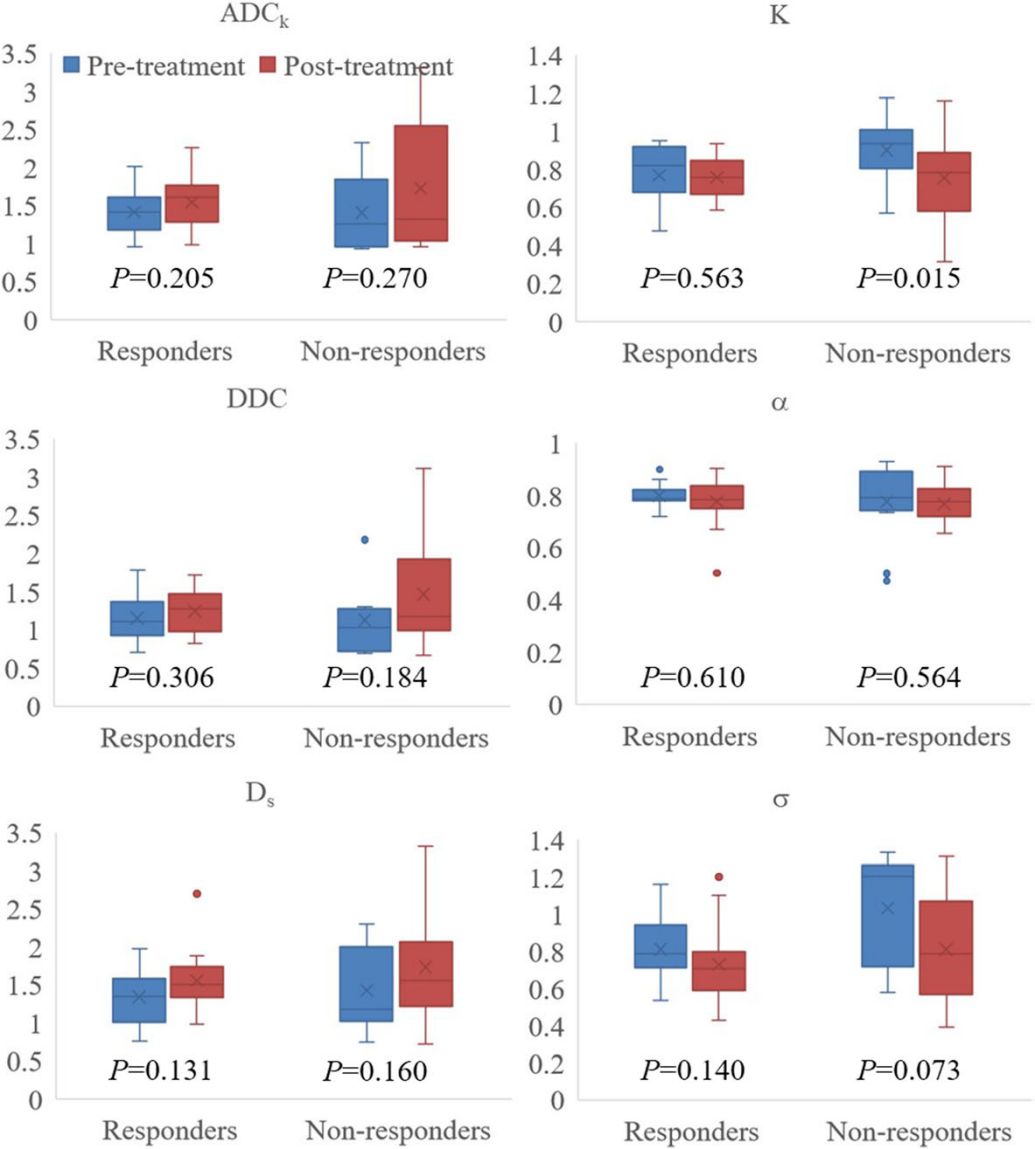
Box plots (median, upper and lower quartiles, maximum and minimum) of the parameters from the non-mono-exponential diffusion models before and after chemotherapy in responders and non-responders

For the non-response group, *K* values were significantly higher at pretreatment, whereas other parameter values did not significantly change. For response group, no parameters presented significant difference before and after chemotherapy (Fig. 2).

### Comparison of parameters between responders and non-responders

**Fig. 3:**
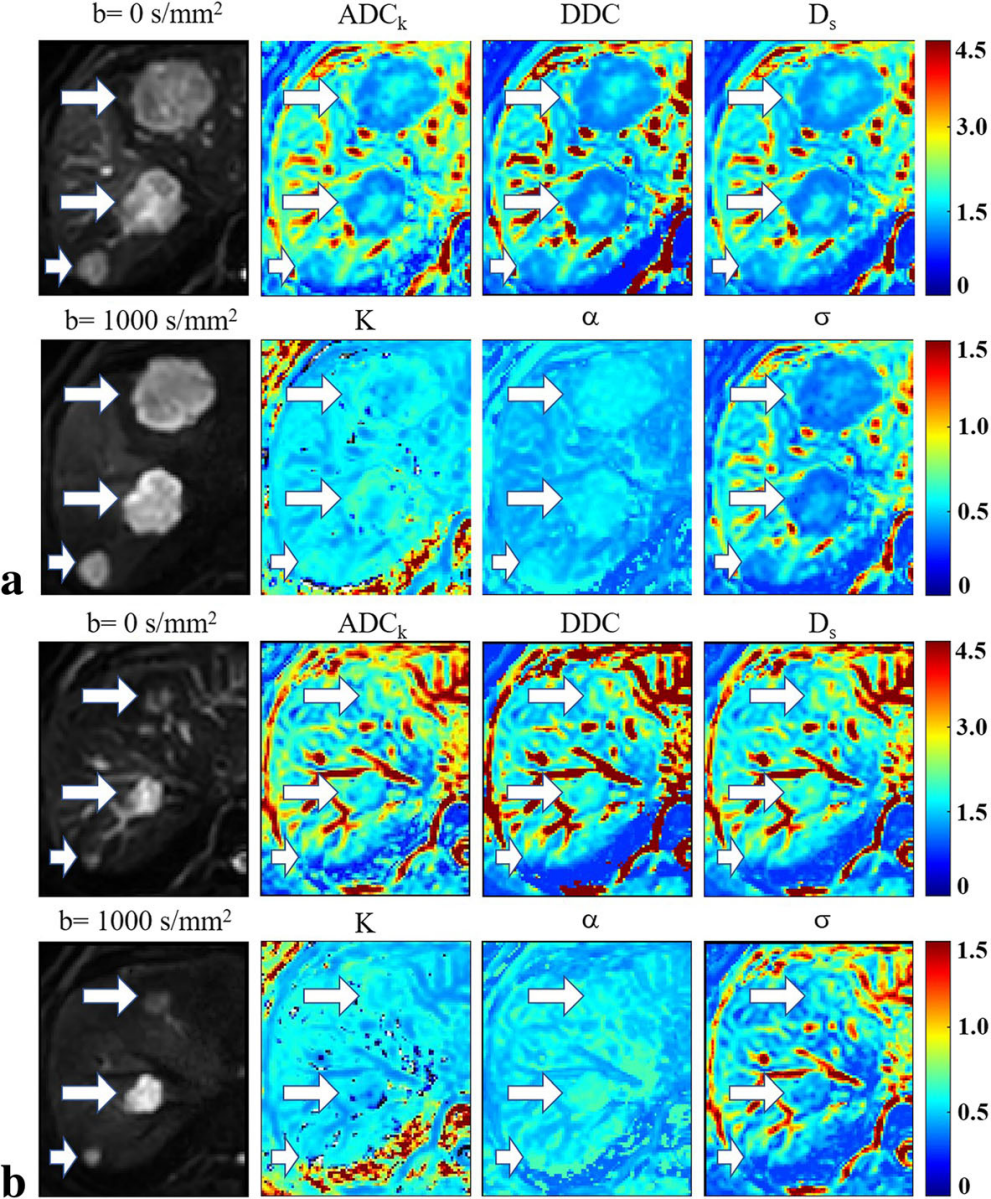
A 47-year-old man with colorectal cancer and liver metastases who was enrolled into the respond group. Images in group **a** are the diffusion-weighted images corresponding to *b* = 0 and 1000 s/mm^2^ as well as the maps of the non-mono-exponential diffusion-related parameters, namely, *ADC*_*k*_, *K*, *DDC*, *α*, *D*_*s*_ and *σ* before chemotherapy. Images in group **b** are the diffusion-weighted images corresponding to *b* = 0 and 1000 s/mm^2^ as well as the maps of the non-mono-exponential diffusion-related parameters after chemotherapy

**Fig. 4:**
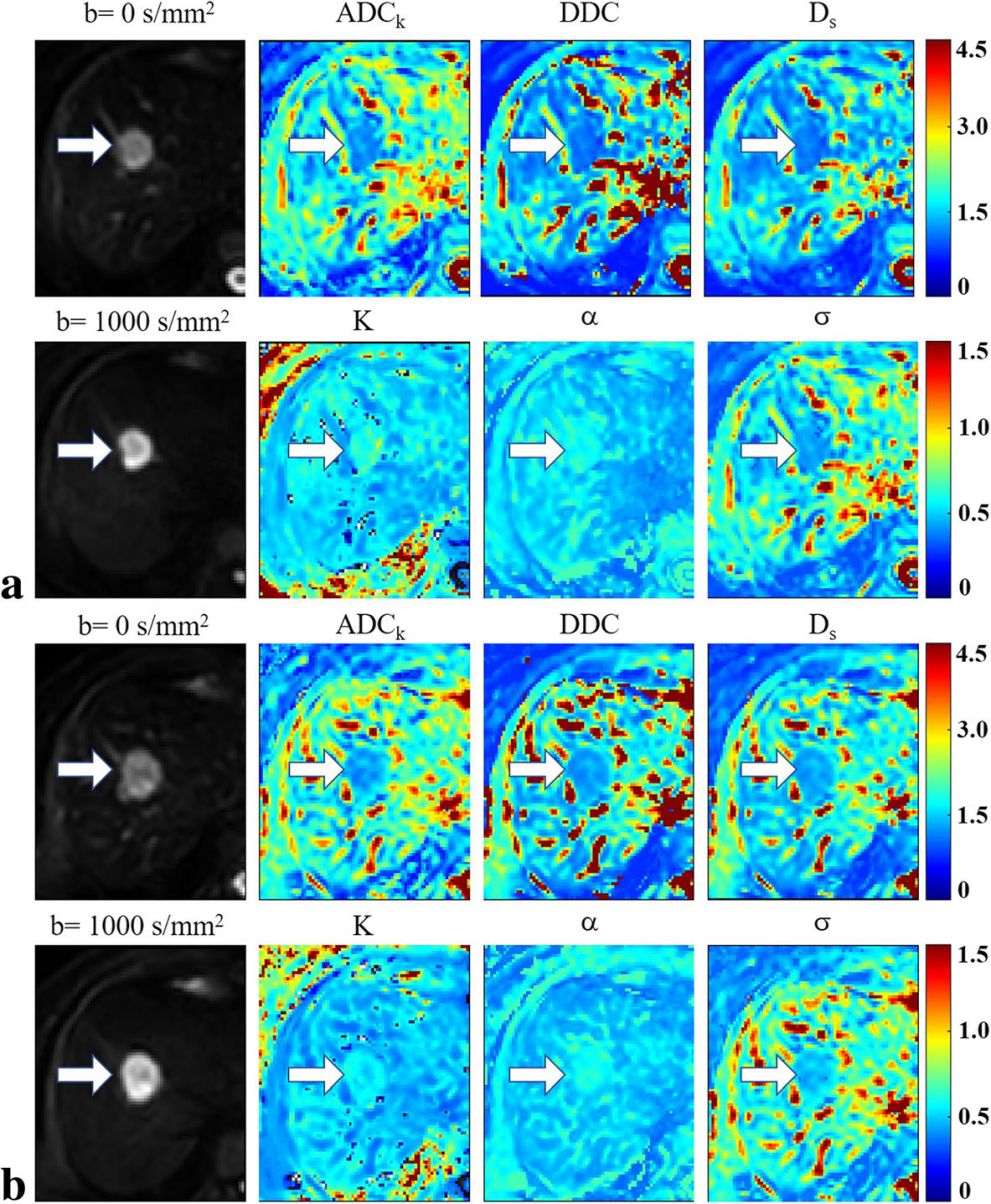
A 62-year-old woman with colorectal cancer and liver metastases who was enrolled into the non-respond group. Images in group **a** are the diffusion-weighted images corresponding to *b* = 0 and 1000 s/mm^2^ as well as the maps of the non-mono-exponential diffusion-related parameters, namely, *ADC*_*k*_, *K*, *DDC*, *α*, *D*_*s*_ and *σ* before chemotherapy. Images in group **b** are the diffusion-weighted images corresponding to *b* = 0 and 1000 s/mm^2^ as well as the maps of the non-mono-exponential diffusion-related parameters after chemotherapy

The response group presented significant lower Pre-*K* and Pre-*σ* values as well as significantly higher Δ*K* and Δ*σ* values compared with the non-response group. No significant differences were observed in terms of other parameters (Table 2, Figs. 3 and 4).

**Table 2:** Comparison of non-mono-exponential diffusion-related parameters before and after chemotherapy between responders and non-responders

	Parameters	Responders (*n* = 17)	Non-responders (*n* = 16)	*P*-value
Pre-treatment	*ADC*_*k*_ (10^− 3^ mm^2^/s)	1.41 ± 0.28	1.40 ± 0.47	0.444
	*K*	0.77 ± 0.15	0.90 ± 0.15	0.015
	*DDC* (10^−3^ mm^2^/s)	1.15 ± 0.27	1.12 ± 0.46	0.382
	*α*	0.80 ± 0.05	0.78 ± 0.13	0.901
	*D*_*s*_ (10^−3^ mm2/s)	1.34 ± 0.37	1.42 ± 0.54	0.817
	*σ*	0.81 ± 0.18	1.03 ± 0.28	0.041
Post-treatment	*ADC*_*k*_ (10^−3^ mm^2^/s)	1.54 ± 0.34	1.72 ± 0.85	0.657
	*K*	0.76 ± 0.11	0.75 ± 0.20	0.929
	*DDC* (10^−3^ mm^2^/s)	1.24 ± 0.26	1.46 ± 0.69	0.817
	*α*	0.78 ± 0.09	0.77 ± 0.09	0.657
	*D*_*s*_ (10^−3^ mm2/s)	1.56 ± 0.37	1.73 ± 0.70	0.845
	*σ*	0.73 ± 0.19	0.81 ± 0.29	0.363
Variation rate	Δ*ADC*_*k*_ (%)	12.61 ± 32.15	23.61 ± 43.74	0.510
	Δ*K* (%)	2.99 ± 26.25	−16.19 ± 18.23	0.021
	Δ*DDC* (%)	13.00 ± 34.94	34.18 ± 48.35	0.276
	Δ*α*(%)	−2.41 ± 13.65	0.20 ± 11.96	0.986
	Δ*D*_*s*_ (%)	22.31 ± 33.41	29.00 ± 51.01	0.817
	Δ*σ*(%)	−8.42 ± 20.20	−20.76 ± 18.49	0.034

*ADC*_*k*_ apparent diffusion coefficient from the kurtosis model, *DDC* distributed diffusion coefficients

### Diagnostic performance for assessing response to chemotherapy

**Fig. 5:**
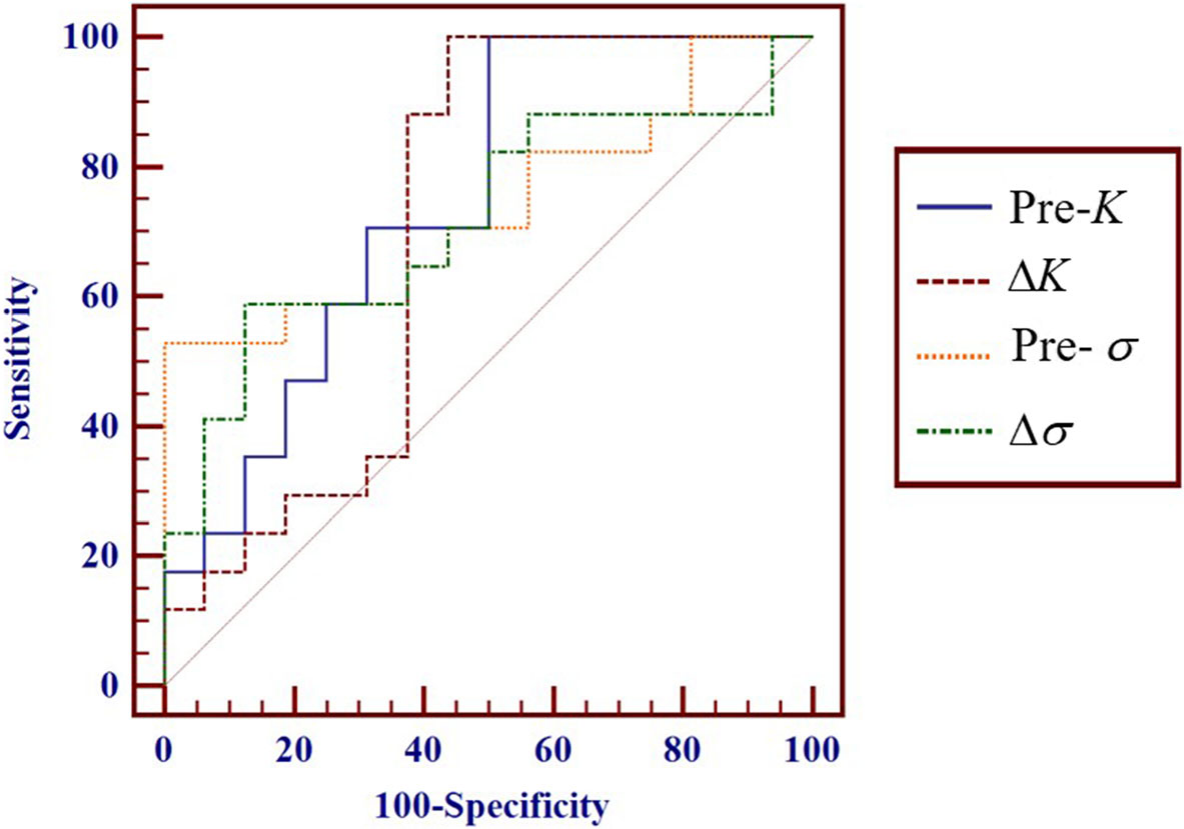
ROC analysis for Pre-*K*, Δ*K*, Pre-*σ* and Δ*σ* in discriminating responders from non-responders

Pre-*K*, Δ*K*, Pre-*σ*, and Δ*σ* located in the area under the curve were 0.746, 0.735, 0.710, and 0.717, respectively. The optimal cutoff value for identifying responders was 0.95 for Pre-*K*, − 1.05% for Δ*K*, 1.16 for Pre-*σ*, − 2.13% for Δσ. The analysis results from the ROC curve are summarized in Table 3 and Fig. 5.

**Table 3:** Diagnostic performance of parameters (assessed by AUCs) derived from the non-mono-exponential models for the detection of respond to chemotherapy

Parameters	AUC(95%CI)	Sensitivity	Specificity	PPV	NPV	Accuracy	Cutoff value
Pre-*K*	0.746 (0.565~0.881)	100.0%	50.3%	68.3%	100.0%	76.3%	0.95
Δ*K* (%)	0.735 (0.553~0.873)	53.4%	100.0%	100.0%	67.5%	76.3%	−1.05
Pre-*σ*	0.710 (0.526~0.854)	100.0%	56.2%	71.1%	100.0%	79.2%	1.16
Δ*σ*(%)	0.717 (0.534~0.859)	59.2%	88.4%	83.4%	67.4%	73.4%	−2.13

*AUC* area under curve, *PPV* positive predictive value, *NPV* negative predictive value

## Discussion

In present study, it was found that the response group had significantly lower *K* and *σ* values at pretreatment (i.e. Pre-*K* and Pre-*σ*) in comparison with the non-response group, which might be helpful for clinicians to predict the response of liver metastases to chemotherapy before treatment begins. At the same time, the absolute values of Δ*K* and Δ*σ* were significantly higher in the non-response group than those in the response group, which to some extent suggests that the heterogeneity of non-responding liver metastases changed even more during chemotherapy. In addition, Pre-*K* presented the highest diagnostic performance, the best sensitivity and relatively high accuracy among all parameters.

These results could also be partly explained by the model itself and by the properties of pathological changes during treatment. The kurtosis, stretched exponential or statistical model could to some extent reflect the heterogeneity of tumor by using the new diffusion-related parameters, such as *K*, *α* and *σ*. When *K* or *σ* is closer to zero or *α* is closer to 1, the DW attenuation signals tend to mono-exponentially decay. Conversely, a higher *K* or *σ* value or a lower *α* value indicates a higher degree of non-mono-exponential signal decay and higher tumorous heterogeneity. In other words, the non-responding liver metastases with higher *K* and *σ* values at pretreatment had more complex microstructure in contrast to the responding ones. This might be due to the distribution of numerous invisible micro-necroses, fibroses and cysts in the non-responding metastases. This pathological feature could result in the deviation of water diffusional behavior fitting by a Gaussian mode and further increase the diffusional heterogeneity of water molecules.

Nevertheless, the phenomenon that the non-responding metastases showed greater change for the *K* and *σ* values before and after chemotherapy compared with responding metastases. The phenomenon was indeed contrary to our general knowledge about the respond of tumors to chemotherapy. In present study, the respond or not of metastases to chemotherapy was determined on basis of RECIST (i.e. the change of tumor size before and after chemotherapy), which was in consistent with previously reported similar studies. At the same time, it was known that the *K* and *σ* values reflected the heterogeneity of tumor. That is to say that the tumorous heterogeneity appeared as significant change after chemotherapy while the tumor itself did not obviously shrinkage. It is very interesting to explore the underlying pathological causes of above the paradoxical phenomenon. But this exceeded the scope of present study.

Additionally, it can be found that there was no statistically significant difference for the heterogeneity parameter *α* between responders and non-responders regardless of before or after chemotherapy. As for the stretched model, the “heterogeneity” refers to the heterogeneity of the exponential decay, rather than the heterogeneity of the diffusion coefficient put by the kurtosis and statistical model [28]. This might be a main reason that *α* did not present significant difference.

In present study, the diffusion coefficients (*ADC*_*k*_, *DDC* and *D*_*s*_) did not show the significant difference between responders and non-responders or between pretreatment and the early treatment period. However, several previous investigations suggested that *ADC* seems to be a promising biomarker for helping to predict early response to chemotherapy of liver metastases. Cui et al. found that an early increase in *ADC*s after initiating chemotherapy in responding hepatic metastases (*P* = 0.002) [29]. Koh et al. measured hepatic metastases using *b* values of 150~500 s/mm^2^ before and after chemotherapy and observed that at pretreatment mean *ADC*s in responding lesions were significantly lower than those of non-responding lesions (*P* < 0.002) [30]. Kim et al. used the intravoxel incoherent motion (IVIM) model to assess early therapeutic response of hepatic metastasis after chemotherapy and observed that the mean *D* values in respond group significantly increases (*P* = 0.012) after the first cycle of chemotherapy [31]. The reason of the disagreement between our results and those from previous studies may be because of the choice of models and *b*-value used. The previous work focused more on the investigation of the mono-exponential or IVIM model in the range of *b* = 0~800 s/mm^2^. These calculated *ADC* or *D* values mixed with the information of tumorous heterogeneity. Nevertheless, the present study used the non-mono-exponential models to fit the DW attenuation signals corresponding to high *b* values (up to 2000 s/mm^2^) and thus eliminated the effect of heterogeneity on the diffusion coefficients (*ADC*_*k*_, *DDC* and *D*_*s*_). Moreover, the significant differences of heterogeneity parameters *K* and σ were observed in present study, which implied that the observed significant change of *ADC* or *D* values in previous studies might have been influenced by changes in tumoral heterogeneity. In fact, it can be also found that the *ADC*_*k*_, *DDC* and *D*_*s*_ values increased to some extent after the first cycle of chemotherapy in this study, but no significant differences were observed. This was probably because chemotherapy not only induces decreased tumor cellularity, but also results in cytotoxic edema [24]. The effect of these pathological changes to water diffusion is bidirectional, so the changes of diffusion coefficients were masked. These results indicated that the separation of diffusion coefficients and heterogeneity parameters was necessary to more exactly reveal the pathological differences or changes of liver metastasis between responders and non-responders or before and after chemotherapy.

This study has several limitations. Firstly, the sample size is relatively small and the study is confined to a single center. So, studies including the larger patient studies on multiple centers are still needed to further validate our findings. In this study, the advantage of non-mono-exponential diffusion parameters were proven based on a small patient population, suggesting that it could be as early clinical biomarkers of response to chemotherapy in patients with liver metastases from CRC. Secondly, this study did not take all timepoints after chemotherapy into account. Thus, the investigations that monitor the parameters derived from the non-mono-exponential models after every cycle of chemotherapy could be done to further obtain more useful information in assessing the chemotherapy response of liver metastases. Finally, a reproducibility analysis of parameters was not performed in present study. But the use of median values for each parameter extracted from ROIs allows obtaining more robust measures.

## Conclusions

The results of this study suggest that the non-mono-exponential DWI might be a potentially valuable clinical tool to predict and determine the response to chemotherapy for patients with liver metastases from CRC. *K* from the kurtosis model might be the most promising biomarker, for it with better diagnostic performance than other parameters in terms of discriminating those tumors being responsive to chemotherapy.

## Data Availability

The datasets used and/or analyzed during the current study are available from the corresponding author on reasonable request.

## Abbreviations

ADC: Apparent diffusion coefficient
AUC: Area under the curve
CR: Complete response
CRC: Colorectal cancer
CT: Computed tomography
DDC: Distributed diffusion coefficient
DW: Diffusion-weighted
DWI: Diffusion-weighted imaging
ICC: Intraclass correlation coefficient
IVIM: Intravoxel incoherent motion
MRI: Magnetic resonance imaging
NPV: Negative predictive value
PD: Progressive disease
PPV: Positive predictive value
PR: Partial response
RECIST: Response evaluation criteria in solid tumors
ROC: Receiver operating characteristic
ROI: Region of interest
SD: Stable disease
TSE: Turbo spin echo
